# Damage Localization and Sensor Layout Optimization for In-Service Reinforced Concrete Columns Using Deep Learning and Acoustic Emission

**DOI:** 10.3390/ma18184406

**Published:** 2025-09-21

**Authors:** Tao Liu, Aiping Yu, Zhengkang Li, Menghan Dong, Xuelian Deng, Tianjiao Miao

**Affiliations:** 1 School of Civil Engineering, Guilin University of Technology, Guilin 541004, China; 2120230888@glut.edu.cn (T.L.); 2120240879@glut.edu.cn (Z.L.); dmh032986@163.com (M.D.); 2017087@glut.edu.cn (X.D.); 1020230752@glut.edu.cn (T.M.); 2 Guangxi Key Laboratory of Green Building Materials and Construction Industrialization, Guilin University of Technology, Guilin 541004, China

**Keywords:** in-service rc columns, acoustic emission, k-means clustering, bp neural network, rbf neural network, svr, sensor layout

## Abstract

As the main load-bearing components of engineering structures, regular health assessment of reinforced concrete (RC) columns is crucial for improving the service life and overall performance of the structures. This study focuses on the health detection problem of in-service RC columns. By combining deep learning algorithms and acoustic emission (AE) technology, the AE sources of in-service RC columns are located, and the optimal sensor layout form for the health monitoring of in-service RC columns is determined. The results show that the data cleaning method based on the k-means clustering algorithm and the voting selection concept can significantly improve the data quality. By comparing the localization performance of the Back Propagation (BP), Radial Basis Function (RBF) and Support Vector Regression (SVR) models, it is found that compared with the RBF and SVR models, the MAE of the BP model is reduced by 7.513 mm and 6.326 mm, the RMSE is reduced by 9.225 mm and 8.781 mm, and the R^2^ is increased by 0.059 and 0.056, respectively. The BP model has achieved good results in AE source localization of in-service RC columns. By comparing different sensor layout schemes, it is found that the linear arrangement scheme is more effective for the damage location of shallow concrete matrix, while the hybrid linear-volumetric arrangement scheme is better for the damage location of deep concrete matrix. The hybrid linear-volumetric arrangement scheme can simultaneously detect damage signals from both shallow and deep concrete matrix, which has certain application value for the health monitoring of in-service RC columns.

## 1. Introduction

Reinforced concrete (RC) has been widely used in construction and engineering because of its high strength and durability. RC components possess inherent flaws prior to their manufacturing or placement into service [1,2]. During the structure’s operational life, these latent defects are subject to a multitude of influencing factors [3]. Consequently, they may evolve into macroscopic cracks, ultimately compromising both the safety and long-term durability of the building structure [1]. The implementation of structural health assessments for primary load-bearing components is imperative. Such proactive evaluations facilitate the timely formulation of targeted intervention strategies and the execution of necessary maintenance prior to the onset of significant structural damage [4]. Consequently, this practice substantially mitigates the economic losses and potential casualties resulting from structural failures.

Non-destructive testing (NDT) is a technique that assesses the integrity or damage of materials and structures without damaging them. Non-destructive testing techniques commonly used for structural damage assessment include acoustic emission (AE), ultrasonic testing (UT) and radiographic testing (RT) [5]. Under the action of force, deformation or fracture occurs inside the material, and when energy is released, instantaneous elastic waves are produced, a phenomenon known as AE [6]. Compared with other NDT techniques, a significant advantage of AE testing resides in its ability to provide real-time, dynamic monitoring of internal damage locations. This capability stems from the fact that the detected signals are generated by the structure or material itself when abnormalities occur [7]. Consequently, AE testing enables continuous assessment of structural integrity during operation. The inspection results can closely approximate the actual internal damage within the test specimen, thereby providing a reliable representation of its structural condition. Owing to this accuracy, the method is extensively employed in the examination of various materials and structures, including mechanical bearings [8], railway substructures [9], metals [10], rocks [11], ceramics [12], and concrete [13].

AE source localization represents a fundamental capability and primary objective of AE-based structural health monitoring. This technique enables the precise identification of internal damage or crack propagation within RC materials in the form of spatial coordinates. The traditional AE source localization algorithm is based on time difference of arrival and the material homogeneity assumption (assuming the AE wave travels at a fixed speed in the material) time difference location [14]. It should be emphasized that AE signals are almost unable to propagate through air when traveling within RC materials [15]. As a result, the waveform undergoes considerable refraction, reflection, and diffraction. Furthermore, the RC medium absorbs a portion of the signal energy, leading to significant alterations in wave velocity. To improve the localization accuracy, some studies have modified the time difference positioning method [16,17], but mostly based on the modified wave velocity. Although some results have been achieved, it is still difficult to detect the actual structure because the attenuation of the signal in the RC structures is difficult to ignore.

The application of deep learning (DL) to AE source localization has been demonstrated to effectively mitigate the adverse effects of material anisotropy, boundary reflections, and obstacles within the propagation path [18]. This capability stems from the intrinsic strength of DL models in modeling and interpreting complex, non-linear wave propagation phenomena [19]. Ai et al. [20] proposed a convolutional neural network (CNN) based on weighted set regression to determine the two-dimensional coordinates of damage locations, addressing detection challenges in nuclear fuel storage facilities. The method was validated using large-scale steel plate specimens, confirming its practical effectiveness. Cheng et al. [21] developed an integrated methodology for AE source localization in steel–concrete composite beams, which combines artificial neural networks with finite element simulation. This approach enables effective AE source identification in I-beams and improves the potential for designing and optimizing AE-based techniques for practical structural inspection applications. Jierula et al. [22] addressed the structural health inspection of in-service RC columns through manual impact excitation at AE source locations. This approach, integrated with neighborhood component analysis, support vector machines (SVM), and BP (Back Propagation) neural networks, achieved accurate damage classification and coordinate localization in RC columns. In a separate study, Vy et al. [23] proposed an AE signal feature extraction method based on the continuous wavelet transform and developed a dedicated CNN model. The proposed framework was subsequently validated on both panel and cube specimens, confirming its effectiveness in AE signal interpretation. The DL-based AE source localization technique is of great research value because it can not only improve localization accuracy, but also solve some problems that traditional methods cannot solve.

Building upon previous research, this study evaluates the efficacy of BP Neural Networks, Radial Basis Function (RBF) Neural Networks, and Support Vector Regression (SVR) in AE source localization for in-service RC columns. A data cleansing methodology based on the k-means clustering algorithm and a voting mechanism is proposed to enhance localization accuracy. Furthermore, the influence of various sensor arrangement strategies on model performance is investigated, leading to the identification of an optimal sensor configuration scheme for damage localization in in-service RC columns.

## 2. Methodology

### 2.1. Experimental Design

**Figure 1:**
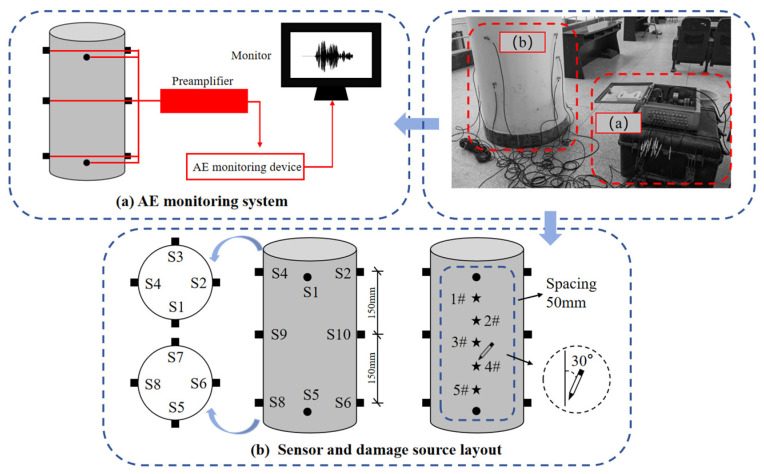
Test System.

The study selected the structural column between the third and fourth floors of teaching building at Guilin University of Technology as the test subject. The column is a cylinder, approximately 3900 mm high, with a radius of approximately 300 mm. The Pencil Lead Break Test (PLBT) is a widely adopted method for generating simulated AE signals. It offers notable advantages including consistent signal stability, a high degree of reproducibility, and straightforward implementation. To simulate actual damage conditions in RC columns, an artificial AE signal was generated in accordance with the standard “Method for AE Detection and Result Evaluation of Metal Pressure Vessels” [24] and “Standard Guide for Determining the Reproducibility of Acoustic Emission Sensor Response” [25]. Specifically, a 0.5 mm diameter HB-grade mechanical pencil was used with its lead extended to 3 mm in length. The lead was then fractured at a 30° angle relative to the surface of the beam. Five damage locations were set on the column, numbered 1# to 5#, and the distance between sensor 1 and the damage locations was regarded as the actual damage location, that is, the damage locations corresponding to 1# to 5# were 50 mm, 100 mm, 150 mm, 200 mm and 250 mm, respectively, and the sensor numbers were S1 to S10. For each damage location, repeat the PLB 30 times. The detailed PLB locations and sensor layout are shown in Figure 1.

### 2.2. Test Equipment

The AE detection equipment used in the experiment was the Sensor Highway3, a third-generation, 16-channel fully digital system manufactured by Physical Acoustics Corporation, USA. This system consists of a preamplifier, a filter, signal acquisition and processing components, and sensors. Set the preamplifier gain to 26 dB. The ambient noise at the test site was tested, and it was found that the amplitude of the ambient noise was all below 30 dB. Therefore, setting the threshold of the AE detection equipment to 35 dB could filter out almost all the ambient noise. At the same time, a PK15I narrowband resonant sensor from the same company was selected. The resonant frequency is 150 kHz and the sensitivity value is greater than 70 dB. Detailed test parameter settings are shown in Table 1.

### 2.3. Test Procedure

(1) Before the test, mark the order and position of the 10 sensors on the RC column.

(2) After the instrument connection is completed, use hot melt adhesive as the coupling agent to adhere the AE sensor to the surface of the RC column. Use the AST function to check the coupling to ensure that the sensor is well coupled to the sample surface.

(3) The threshold of the acoustic emission system was set to 35 dB, and the preamplifier gain was configured to 26 dB. Proper configuration of time parameters is critical for accurate signal acquisition. According to conventional practice, the hit definition time (HDT) should be twice the peak definition time (PDT), and the hit lock time (HLT) should be marginally longer than the HDT. Accordingly, the PDT, HDT, and HLT were assigned values of 250 μs, 500 μs, and 600 μs, respectively.

(4) Collect the PLB signal. While performing PLB signal, turn on the AE device and repeat the PLB 30 times for each damaged position.

### 2.4. K-Means Clustering Algorithm

K-means clustering is a type of unsupervised learning that iteratively divides data into “k” clusters so that data points within the same cluster are as similar as possible and data points between different clusters are as different as possible. Through iterative optimization, the algorithm gradually reduces the distance between the data points within the cluster and the cluster center. The core mechanisms of the algorithm’s operation include initializing cluster centers, allocating data points, updating cluster centers, and iterative optimization. The k-means clustering is explained in detail in the literature [26]. The distance from the data point to the cluster center is calculated using the Euclidean distance formula as follows:

(1) $$D(x,\mu )=\sqrt{\sum_{i=1}^{n}(x-\mu_{i}{)}^{2}}$$

where *n* represents the number of clusters; *x* is the data point in the dataset; *μ_i_* represents the cluster center of the *i*-th cluster.

### 2.5. BP Neural Network

**Figure 2:**
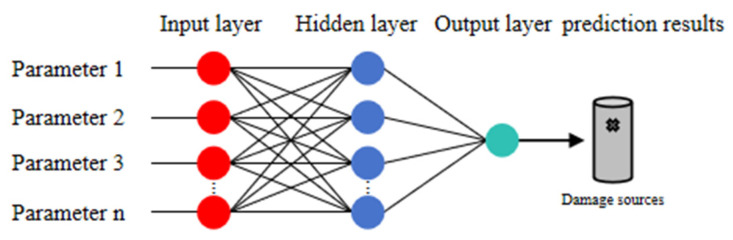
Topology of BP Neural Network.

BP neural network is a supervised DL algorithm that mainly consists of an input layer, a hidden layer, and an output layer [27]. The input layer takes input data from outside, the hidden layer performs feature transformation on the data, and the output layer is responsible for outputting the calculation results. It is notable that the calculations of the BP neural network are carried out in the forward direction, while the errors are calculated through backpropagation. The purpose of the error calculation is to adjust the weights and thresholds of the neural network in order to make the output result more accurate. The algorithm stops when the loss function converges or reaches the maximum number of iterations. The BP neural network is explained in detail in the literature [28]. The BP neural network was employed for AE source localization and demonstrated promising accuracy [22]. The basic principle of the BP neural network is shown in Figure 2.

### 2.6. Radial Basis Function Neural Network

Like BP neural networks, RBF neural networks also consist of input layers, hidden layers, and output layers. The difference is that BP neural networks typically use the ReLu function as the activation function for the hidden layer, while RBF neural networks use the RBF as the activation function. Correspondingly, the neurons in an RBF neural network are also called RBF neurons. The output layer performs a linear weighted combination of the processing results of the hidden layer to obtain the final predicted value. In this process, the RBF neural network achieves a non-linear mapping of the input data through local response characteristics. Compared with BP neural networks, RBF neural networks are simpler in structure and faster in training speed. The integration of the RBF neural network with AE technology demonstrates significant potential in predicting the remaining useful life of mechanical rotors [29]. The calculation formula of the RBF is as follows:

(2) $$V(x,c_{i})=\exp(-\frac{||x-c_{i}|{|}^{2}}{2\sigma_{i}^{2}})$$

where *x* is the input vector, *c_i_* is the center vector of the *i*-th hidden layer neuron, and *σ_i_* is the width parameter of the *i*-th hidden layer neuron.

### 2.7. Support Vector Regression Prediction

SVR is an extension of SVM. SVM is often used for classification tasks, while SVR is used for regression tasks. The core idea of SVM is to find an optimal hyperplane (decision boundary) that maximizes the intervals between different classes. Similarly to SVM, SVR aims to identify an optimal hyperplane within the feature space. This hyperplane acts as a regression function that minimizes deviation from the data points while permitting predictions to lie within a predefined tolerance margin. Notably, errors within this margin are not penalized, thereby enhancing the model’s flexibility and generalization capability. The range of this allowable error is controlled by a parameter *ε*, so the loss function here is called the ε-insensitive loss function. The SVR model is explained in detail in the literature [30]. The integration of AE technology with the SVR model has been employed to classify crack types induced during concrete fracture processes [31]. The loss function is calculated as follows:

(3) $$L_{\varepsilon }(y_{i},f(x_{i}))=max(0,|y_{i}-f(x_{i})|-\varepsilon )$$

where *f*(*x_i_*) is the predicted value, *y_i_* is the true value, and *ε* is the error control parameter.

## 3. Results and Analysis

During the initial phase of data processing, a comprehensive set of AE parameters was extracted to fully characterize both the time-domain and frequency-domain attributes of the signals. The parameters primarily include channel number, rise time, ringing counts, energy, duration, amplitude, average frequency, counts to peak, reverberation frequency, initiation frequency, frequency centroid, peak frequency, signal strength, and absolute energy. For the convenience of subsequent data analysis, all parameters were normalized.

### 3.1. AE Data Cleaning Based on k-Means Clustering

Studies have shown that the waveform of AE signals is distorted when propagating in an RC structure [32]. Due to the reflection and diffraction of the AE signal, the propagation path of the wave is also uncertain. It should be emphasized that signal distortion increases with propagation distance. Furthermore, the availability of a high-quality dataset is critical for effectively training DL models, as it directly influences their predictive performance [33]. The distances from different sensors to the damage localization are not the same. Consequently, during the acquisition of PLB signals, the waveforms captured by individual sensors may exhibit variations in content and characteristics. Such inconsistency adversely affects the accuracy and reliability of damage source localization. To minimize the problem of important signal information loss caused by waveform distortion as much as possible, the k-means algorithm and the voting selection concept are introduced for data cleaning. Specifically, for all signals of a certain damage position, the clustering algorithm is first used for category division, with each damage signal corresponding to one category. Then, a vote is taken to determine whether this group of signals (10 signals collected in the same PLB test) are high-quality signals.

**Figure 3:**
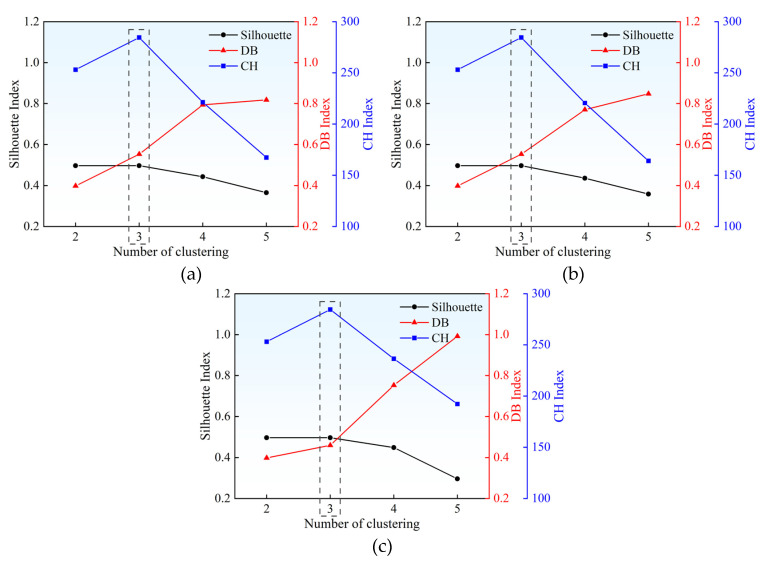
Evaluation of the optimal number of clusters: (**a**) The number of clusters at the damage location 1; (**b**) The number of clusters at the damage location 3; (**c**) The number of clusters at the damage location 5.

Before performing k-means clustering, an assessment of the optimal number of clusters is conducted. The metrics commonly used to assess the number of clusters include the Silhouette Coefficient Index, the Davies-Bouldin Index (DB), and the Calinski-Harabasz index (CH) [34]. The smaller the DB value, the larger the silhouette coefficient and CH values, the better the clustering effect. For each damage location, set the number of clusters to 2 to 5 and calculate the three indicators, respectively. The calculation results of the contour coefficients, DB, and CH values for damage location 1, damage location 3, and damage location 5 are shown in Figure 3. For each damage location, the consistent clustering into three groups indicates the presence of approximately three distinct patterns within the PLB signals. This phenomenon can likely be attributed to several factors, including electromagnetic instrument noise, structural vibration noise, and signal attenuation-induced information loss.

**Figure 4:**
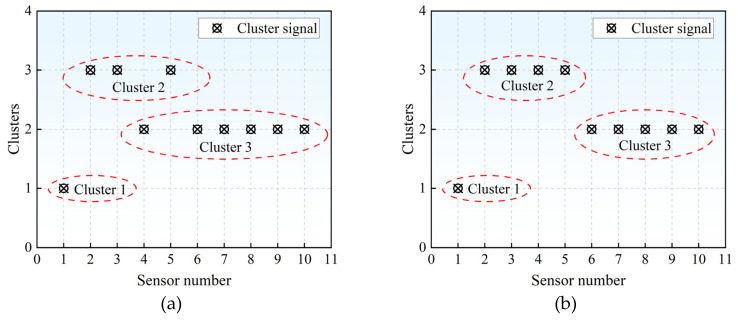
Voting display of the k-means clustering algorithm: (**a**) Effective voting results; (**b**) Invalid voting results.

Set the number of clusters to 3 for all the signals collected at each damage location and perform k-means clustering, respectively. The results of the clustering are used for the voting selection of high-quality signals. When there are six or more signals in each group belonging to the same category, it is considered that the group of signals has higher credibility and is less affected by external interference. The core concept underlying the voting selection mechanism is visually summarized in Figure 4. To evaluate the reliability of the voting-derived results, the original dataset will subsequently undergo training and testing procedures. The predictive performance and robustness of the method will be assessed by analyzing the resulting errors on the test set. The original dataset is named Dataset 1.

### 3.2. Locating AE Sources Using Multiple Deep Learning Methods

The setting of hyperparameters for the model is important. In the BP neural network, the number of hidden layers is set to 14 by calculating the Mean relative error (MRE), the number of iterations is 1000, the target error is 0.000001, and the learning rate is 0.01. In the RBF neural network, the number of neurons in the hidden layer is autonomously determined by the newrbe function, and the network expands at a rate of 100. In the SVR model, the kernel function type is radial basis kernel, the kernel function parameter is 0.8, the penalty factor is set to 4.0, and the loss function parameter is 0.01.

A significant imbalance in the size of different data categories may lead the algorithm to prioritize features associated with the majority class during training, thereby overlooking patterns representative of minority samples. To prevent such problems, 10 sets of signals for each damaged position were selected to construct the dataset. The dataset contains 500 signals, each of which has 14 features and 1 output, the result of which is the distance between sensor 1 and the damage location. Divide the dataset into a training set and a test set in an 8:2 ratio. The training set consists of 400 sets of data, and the test set consists of 100 sets of data. Input the training set into BP, RBF, and SVR models for training, and use the test set to test the positioning performance of the models. To compare the performance of the three models, goodness-of-fit R^2^, mean absolute error (MAE), and root mean square error (RMSE) were introduced as evaluation metrics for the models. The calculation formulas for the three evaluation metrics are as follows:

(4) $$R^{2}=1-\frac{\sum_{i=1}^{n}(b_{i}-b_{k}{)}^{2}}{\sum_{i=1}^{n}(b_{k}-b{)}^{2}}$$

(5) $$MAE=\frac{1}{n}\sum_{i=1}^{n}|b_{i}-b_{k}|$$

(6) $$RMSE=\sqrt{\frac{1}{n}\sum_{i=1}^{n}{\left(b_{i}-b_{k}\right)}^{2}}$$

where *b_i_* represents the actual value, *b_k_* represents the predicted value, *b* is the mean of the actual value, and *n* is the sample size.

The performance evaluation indicators of the three models obtained through calculation are shown in Table 2. All the indicators of the BP neural network have achieved good results. Compared with the RBF and SVR models, MAE decreased by 7.513 mm and 6.326 mm, RMSE decreased by 9.225 mm and 8.781 mm, and R^2^ increased by 0.059 and 0.056. It can be seen from this that BP neural networks can achieve good results in AE source localization. When dataset 1 was input into the BP neural network for training, it was found that after voting selection, the MAE of the test dataset decreased by 8.95 mm, the RMSE decreased by 12.444 mm, and the R^2^ increased by 0.088, indicating that voting selection was effective.

**Figure 5:**
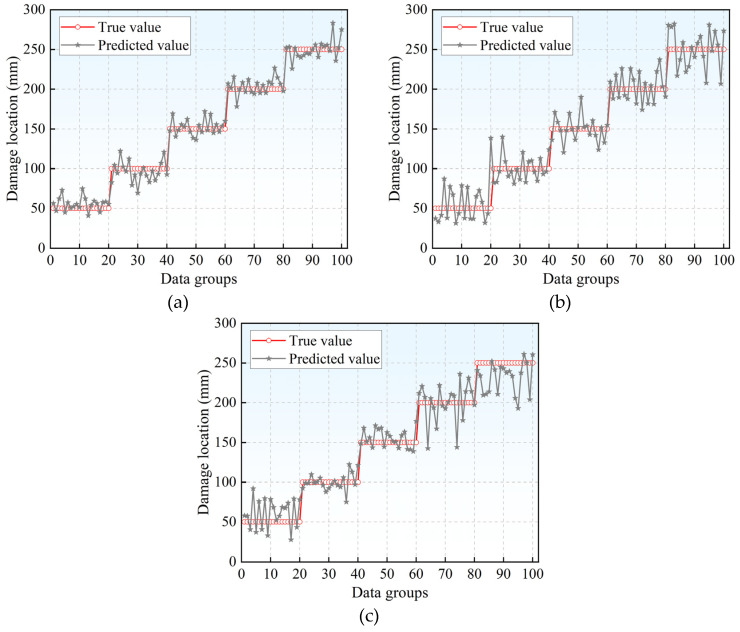
Source localization results for different models: (**a**) BP neural network source localization results; (**b**) RBF neural network source localization results; (**c**) SVR model source localization results.

**Figure 6:**
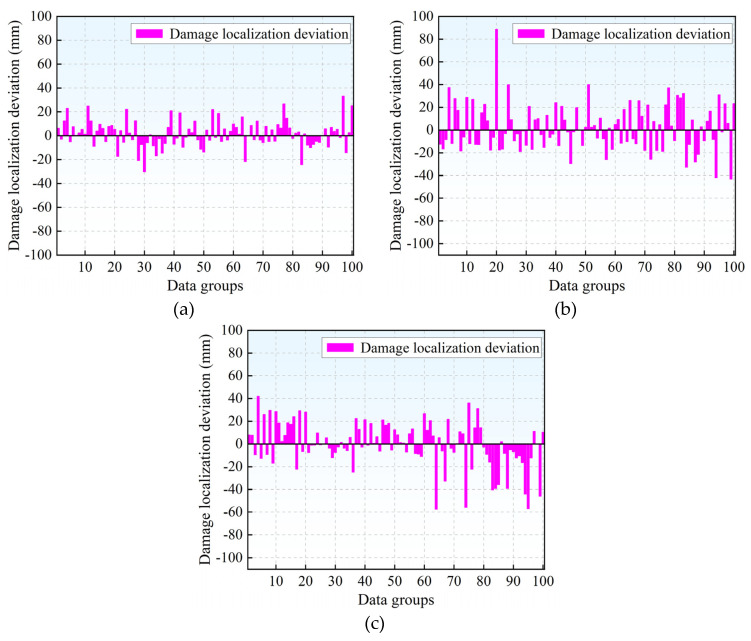
Source localization deviation of different models: (**a**) BP neural network source localization deviation; (**b**) RBF neural network source localization deviation; (**c**) SVR model source localization deviation.

The source localization results and corresponding deviations for the three model test sets are presented in Figure 5 and Figure 6, respectively. Source localization deviation is defined as the discrepancy between the predicted and actual coordinates. Among the evaluated models, the BP neural network exhibits the minimal deviation in localization outcomes and demonstrates the highest accuracy in predicting damage locations. In contrast, both the RBF and SVR models show considerably larger localization errors, which fall outside acceptable thresholds for practical engineering applications.

## 4. Exploration of the Optimal Layout and Quantity of Sensors

The utilization of a 10-sensor array for damage source localization entails significant operational costs. Moreover, a portion of the localization inaccuracies may originate from signals captured by sensors situated at considerable distances from the actual damage source, where signal attenuation and environmental interference are more pronounced. To reduce detection costs and improve localization accuracy, it is necessary to explore the optimal layout form and quantity of sensors.

### 4.1. Layout Schemes for Different Sensor Numbers and Locations

**Figure 7:**
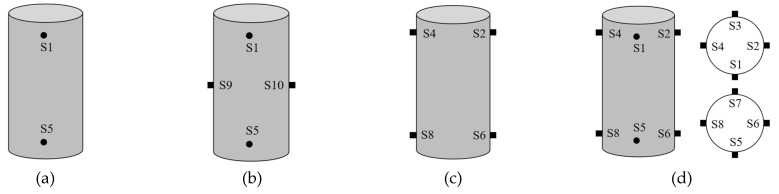
Sensor layout scheme: (**a**) Layout scheme 1; (**b**) Layout scheme 2; (**c**) Layout scheme 3; (**d**) Layout scheme 4.

Four sensor layout schemes are proposed in this study. The first one is a linear arrangement, where the damage source and sensors are positioned on the same straight line, with a total of 2 sensors laid out. The second type is hybrid linear-volumetric arrangement, in which sensor 1 and sensor 5 are in linear arrangement, while sensor 9 and sensor 10 are in volumetric arrangement. The third scheme is a volumetric arrangement with a total of 4 sensors placed on both sides of the damage source. The fourth scheme involves 8 sensors arranged in a circular (ring-shaped) manner. The specific layout forms and positions of sensors in all schemes are illustrated in Figure 7.

### 4.2. Research on Positioning Performance of Each Scheme

The reliability of the BP neural network for locating damage was confirmed in Section 2, so this section uses the BP neural network to test the localization performance of each scheme. The training set and the test set are divided in an 8:2 ratio. Scheme 1 contains 100 sets of data, with 80 sets of training and 20 sets of testing. Scheme 2 and scheme 3 each contain 200 sets of data, with 160 sets of training and 40 sets of testing. Scheme 4 contains 400 sets of data, 320 sets of training and 80 sets of testing. Likewise, the localization results of different schemes were evaluated by R^2^, MAE, and RMSE.

**Figure 8:**
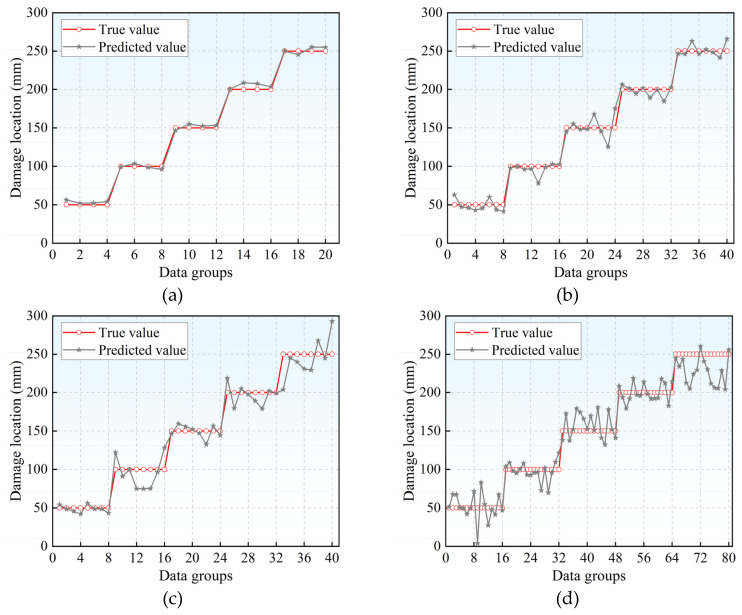
Source localization results of different sensor layout schemes: (**a**) The source localization result of the layout scheme 1; (**b**) The source localization results of the layout scheme 2; (**c**) The source localization results of the layout scheme 3; (**d**) The source localization results of the layout scheme 4.

**Figure 9:**
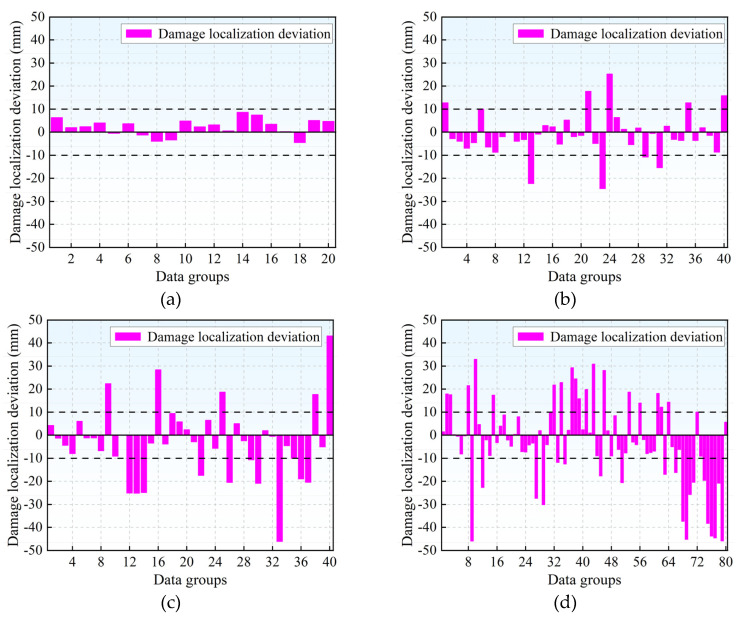
Source localization deviations of different sensor layout schemes: (**a**) Source localization deviation of the layout scheme 1; (**b**) Source localization deviation of the layout scheme 2; (**c**) Source localization deviation of the layout scheme 3; (**d**) Source localization deviation of the layout scheme 4.

The R^2^, MAE and RMSE of different sensor layout schemes are shown in Table 3. The MAE of scheme 1 is only 3.656 mm, indicating that the overall error of the localization results is small. RMSE is at its minimum and R^2^ is at its maximum. The localization error of scheme 2 was greater than that of scheme 1, but it was smaller compared to the layout of 10 sensors. The errors in schemes 3 and 4 are larger and cannot accurately locate the source of damage. The localization results and deviations of the four layout schemes are shown in Figure 8 and Figure 9. The localization results of schemes 1 and 2 fluctuate less compared to the actual source location, while those of schemes 3 and 4 fluctuate more. In Figure 9, a boundary with a localization deviation of ±10 mm is plotted. The localization deviations of scheme 1 are all within 10 mm, 75% of those of scheme 2 are within 10 mm, 60% of those of scheme 3 are within 10 mm, and 51.25% of those of scheme 4 are within 10 mm.

For clarity in subsequent discussion, we define two terms: the shallow concrete matrix (closer to the surface) and the deep concrete matrix (farther from the surface). It is worth noting that although scheme 1 achieved relatively good results, the signal might only propagate in the shallow concrete matrix close to the straight line connecting the sensor and the location of the PLB. This results in less signal attenuation and less loss of important information. The simulated damage was carried out on the surface of the column, but in reality, most damages occur internally. This means that scheme 1 may not achieve ideal results for detecting damages in the deep concrete matrix. Although scheme 2 has a larger error compared to scheme 1, it is still smaller than the original positioning error (Table 2). This hybrid configuration, which integrates both linear and volumetric sensor arrangements, is designed to enhance the detection of damage across both superficial and deep-seated regions within the RC column. The approach accounts for the full signal propagation path (from the excitation event through the heterogeneous concrete medium until reception by the sensors), thereby improving localization accuracy in complex structural environments. Signal propagation within RC structures exhibits a non-linear nature, entailing complex phenomena such as repeated reflection, scattering, and diffraction. Consequently, the signals ultimately captured by the sensors represent composite waveforms resulting from the superposition of multiple wave modes. Scheme 2 is both economical and accurate, reducing the cost by 60% compared to the arrangement of 10 sensors. In summary, the sensor arrangement in scheme 1 is more suitable for detecting damages in the shallow concrete matrix, while scheme 2 is more appropriate for damages occurring in the deep concrete matrix.

## 5. Discussion

To explore the source of the error, a set of signals for each damage location was extracted for analysis. It should be noted that all the extracted signals were selected by the k-means clustering algorithm through voting. The indicators commonly used to measure signal strength mainly include AE energy [35] and AE amplitude [36]. AE energy refers to the measured area under the envelope of the detected signal, and AE amplitude is the maximum voltage value of the AE waveform.

**Figure 10:**
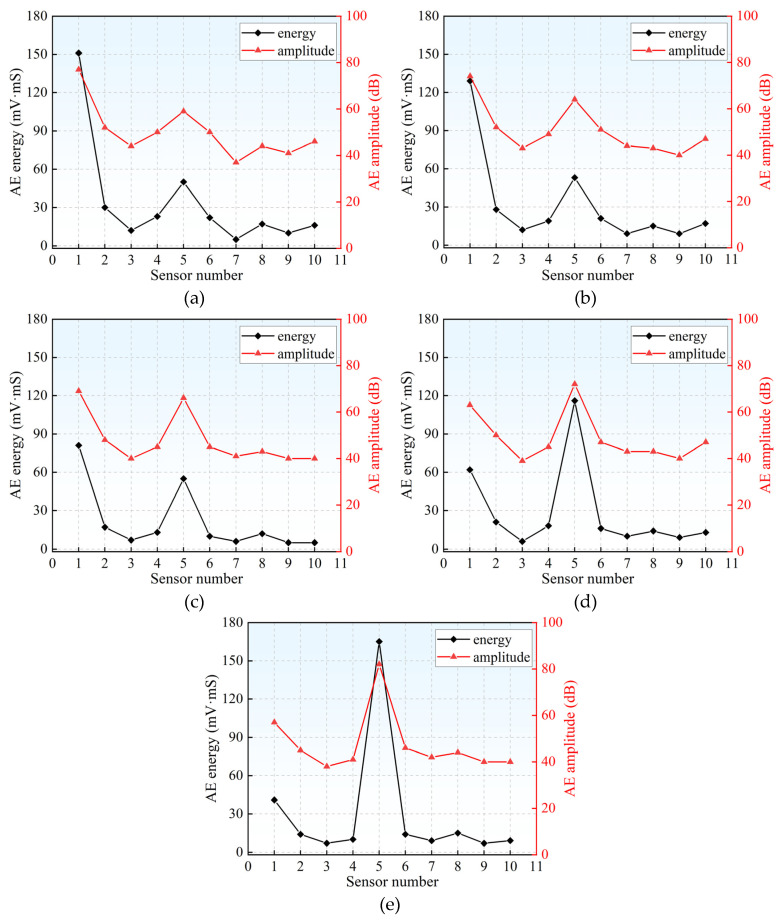
Analysis of signal characteristics at different damage locations: (**a**) Damage source 1; (**b**) Damage source 2; (**c**) Damage source 3; (**d**) Damage source 4; (**e**) Damage source 5.

The energy and amplitude distribution of the signals received by 10 sensors at different damage locations is shown in Figure 10. Regardless of the damage location, Sensors 1 and 5 consistently record the highest energy and amplitude magnitudes. This observation confirms the hypothesis that, under a linear sensor arrangement, AE signals propagate primarily through the superficial layer of the concrete matrix, where attenuation remains relatively low. It can be noted that the energy and amplitude received by sensors 3 and 7 from each damage location are minimal, with an energy of about 5 to 15 mV·mS and an amplitude of about 38 to 45 dB. This observation indicates that the signals received at this location have undergone substantial attenuation, resulting in the loss of critical information pertaining to the damage source. Furthermore, the acquired signals are likely contaminated by structural vibration noise and electromagnetic interference, which collectively constitute a primary contributor to the localization inaccuracy of the model. To avoid such errors, it is recommended to avoid ring-shaped sensor placement during RC column health monitoring. Instead, the sensors should be placed in a linear or hybrid linear-volumetric manner according to scheme 1 or scheme 2, which can improve localization accuracy and save detection costs.

It should be noted that this study serves as a preliminary investigation into both the localization of AE sources in in-service RC columns and the corresponding sensor arrangement strategies. Although DL-based methods can achieve high accuracy in locating AE sources within such structures, they place exceptionally high demands on the feature extraction capabilities of the models. According to relevant research, preprocessing raw data can significantly enhance the precision of AE source localization. Effective approaches include, but are not limited to, selecting parameters rich in source information through neighborhood component analysis [22], processing signals via continuous wavelet transform [23], and employing weighted integration of multiple signal processing techniques [20] for improved feature extraction. Certainly, although these processing methods can enhance localization accuracy, they remain computationally complex and demand substantial computational resources. It is also important to note that most current studies validate models primarily through simulated damage scenarios, with limited application to real-world structural health monitoring. Thus, the integration of DL and AE techniques for locating damage in concrete or RC structures remains largely exploratory. If the approximate range of actual AE source locations can be estimated in advance using other methods (such as Bayesian methods), and then combined with the neural network model and the small-scale sensor arrangement pattern proposed in this study, it may be possible to achieve accurate monitoring of actual damage in RC columns. Specifically, once the general area of damage in in-service RC columns is identified, deploying sensors according to the small-scale arrangement pattern proposed in this paper and applying the neural network model for localization may yield improved positioning accuracy.

## 6. Conclusions

The research conducted AE detection on in-service RC columns by simulating damage sources, and established an AE source localization model for in-service RC columns in combination with multiple DL algorithms. The optimal sensor layout scheme for AE source localization of in-service RC columns was explored. The main conclusions are as follows:

(1) A data cleaning method based on the k-means clustering algorithm and the concept of voting selection was proposed. It was verified that this method could significantly improve data quality and increase the accuracy of the AE source localization model.

(2) By comparing the localization performance of the BP, RBF and SVR models, it was found that the BP model achieved good results in all indicators. Compared with the RBF and SVR models, the MAE of the BP model decreased by 7.513 mm and 6.326 mm, the RMSE decreased by 9.225 mm and 8.781 mm, and the R^2^ increased by 0.059 and 0.056. BP neural networks can achieve good results in AE source localization.

(3) The optimal sensor layout scheme for in-service RC column AE source localization was explored. For damage sources in the shallow concrete matrix, scheme 1 (linear arrangement) is suitable, with MAE and RMSE of only 3.656 mm and 4.26 mm, R^2^ reaching 0.996, and localization deviations within 10 mm. However, the scheme still has certain limitations for damage detection in the deep concrete matrix. For damage detection in the deep concrete matrix, using scheme 2 (linear-volumetric hybrid arrangement) can yield good results. MAE and RMSE were only 6.943 mm and 9.547 mm, R^2^ reached 0.982, and 75% of the localization deviation was within 10 mm. The scheme is both economical and accurate, with a 60% reduction in cost compared to a 10-sensor setup. At the same time, it can detect damage signals from the shallow concrete matrix, which has some application value for the health detection of in-service RC columns.

## Figures and Tables

**Table 1:** Test Parameter Settings.

Name	Value	Name	Value
Threshold value (dB)	35	Peak definition time (µs)	250
Preamplifier gain (dB)	26	Hit definition time (µs)	500
Sampling frequency (MHz)	1	Hit lock time (µs)	600
Number of sensors	10	-	-

**Table 2:** Error display of different model test sets.

	MAE (mm)	RMSE (mm)	R^2^
BP	8.828	11.435	0.974
RBF	16.341	20.660	0.915
SVR	15.154	20.216	0.918
BP (Dataset 1)	17.778	23.879	0.886

**Table 3:** Error display of different sensor layout schemes.

	MAE (mm)	RMSE (mm)	R^2^
Sensor layout 1	3.656	4.260	0.996
Sensor layout 2	6.943	9.547	0.982
Sensor layout 3	11.868	16.301	0.947
Sensor layout 4	14.441	18.963	0.928

## Data Availability

The original contributions presented in this study are included in the article. Further inquiries can be directed to the corresponding author.
